# Proximal Ulnar Osteotomy as a Treatment for Humeral Intracondylar Fissure in a Shetland Sheepdog

**DOI:** 10.3390/ani13030519

**Published:** 2023-02-01

**Authors:** Stavros Karydas, Alan Danielski

**Affiliations:** 1 The Ralph Veterinary Referral Centre, Marlow SL7 1YG, UK; 2 Department of Veterinary Medicine and Animal Sciences, University of Naples “Federico II”, 80138 Naples, Italy

**Keywords:** HIF, humeral intracondylar fissure, IOHC (incomplete ossification humeral condyle), proximal ulnar osteotomy, humero-anconeal incongruity, spaniels, dog

## Abstract

**Simple Summary:**

Humeral intracondylar fissure (HIF) is a common orthopaedic disease affecting Spaniel breed dogs. Surgical treatment traditionally involved placement of a large transcondylar screw to avoid catastrophic consequences such as a fracture. The reported complication rate related to placement of a transcondylar screw is high and varies between 8 and 59.5%. A recent study describing a cartilaginous lesion present on the caudal aspect of the humerus of spaniel breed dogs with HIF has suggested that humero-anconeal incongruency may be the cause of this abnormal cyclical loading that subsequently leads to stress fracture formation. Theoretically, healing of the HIF could therefore be achieved by performing a proximal ulnar osteotomy and therefore interrupting the abnormal, cyclical loading applied by the anconeal process on the humeral condyle. The purpose of this report is to describe the first case of humeral intracondylar fissure in a Shetland sheepdog and to test our hypothesis that proximal ulnar osteotomy can promote healing of the humeral intracondylar fissure.

**Abstract:**

A seven-month-old male Shetland Sheepdog was presented for assessment of thoracic limb lameness of 3 weeks duration. Orthopaedic examination revealed severe discomfort in elbow extension, bilaterally. CT-scan confirmed presence of a complete humeral intracondylar fissure (HIF), bilaterally, and arthroscopic examination of both elbows confirmed the presence of the cartilaginous lesion previously reported in dogs suffering from HIF. A staged oblique proximal ulnar osteotomy was performed to address the humero-anconeal incongruency believed to be the cause of HIF formation. Orthopaedic examination performed 5 weeks after each surgical procedure confirmed that pain previously present on elbow manipulation had subsided. Follow-up examination performed 8 months after the second surgery revealed the dog to be sound at walking on the thoracic limbs with no discomfort present on elbow manipulation. Repeated CT scan confirmed complete healing of both HIFs. This is the first report documenting the presence of HIF in a Shetland sheepdog and complete healing of both HIFs following a proximal ulnar osteotomy.

## 1. Introduction

Humeral intracondylar fissure is a common cause of thoracic limb lameness in dogs in the United Kingdom and it predisposes them to condylar fractures with minimal or no trauma. This disease has been reported in several breeds, but it is most commonly seen in Spaniel breed dogs [1,2,3,4]. Of those breeds affected with HIF, English springer spaniels appear overrepresented with an incidence of 14%, followed by French Bulldogs (11.1%) and Pugs (11.1%) [1,5]. A clear aetiopathogenesis has not yet been established, but this condition is currently believed to be the result of a stress fracture of the humeral condyle as a result of abnormal cyclic loading of the humeral condyle [6]. Surgical treatment traditionally involved placement of a large transcondylar screw to avoid catastrophic consequences such as a fracture. The reported complication rate related to placement of a transcondylar screw is high and varies between 8 and 59.5% [7,8].

A recent study describing a cartilaginous lesion present on the caudal aspect of the humerus of spaniel breed dogs with HIF has suggested that humero-anconeal incongruency may be the cause of this abnormal cyclical loading that subsequently leads to stress fracture formation [9]. Theoretically, healing of the HIF could therefore be achieved by interrupting the abnormal, cyclical loading applied by the anconeal process on the humeral condyle. Proximal ulnar osteotomy (PUO) is a well-known technique used to improve elbow incongruency as it allows proximal translation of the proximal ulnar segment as a result of the upward pull of the triceps muscle [10,11].

The purpose of this report is to describe the first case of humeral intracondylar fissure in a Shetland sheepdog. Moreover, we hypothesised that proximal ulnar osteotomy could lead to bone healing of the humeral intracondylar fissure.

## 2. Materials and Methods

A 7-month-old, 5.5 kg, male Shetland Sheepdog was presented for assessment of thoracic limb lameness. Orthopaedic examination demonstrated severe discomfort in extension of both elbows and none in flexion. Computer tomography examination (GE Revolution, GE Healthcare, Chalfont St Giles, UK) of both elbows was performed under deep sedation using 0.6 mm slices thickness. A 3-D multiplanar reconstruction demonstrated a complete intracondylar hypoattenuating line, extending from the articular surface to the supratrochlear foramen, surrounded by hyperattenuating sclerotic bone. This was bilateral and compatible with HIF.

### Surgical Treatment

**Figure 1:**
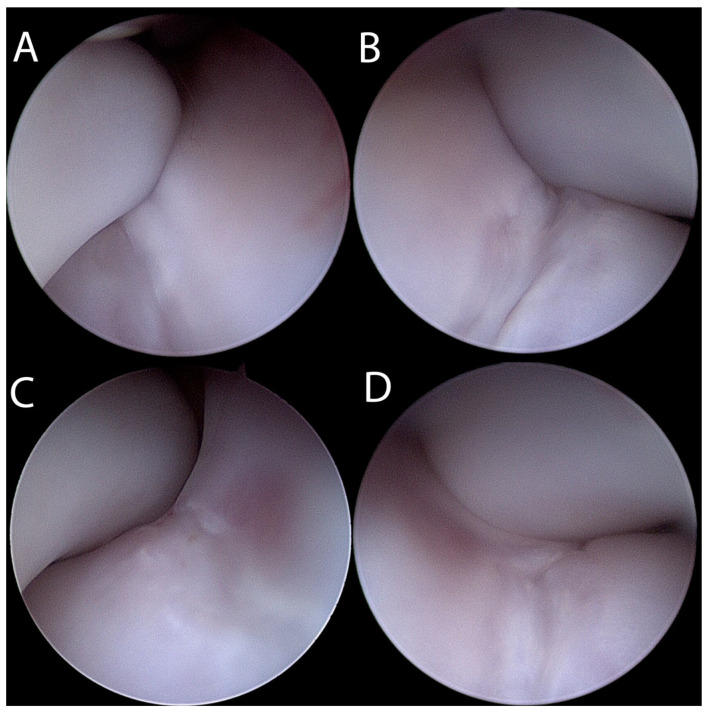
Arthroscopic images of the left elbow (**A**,**C**) and of the right elbow (**B**,**D**) using a caudal portal. Note the positioning of the anconeal process matching the cartilaginous lesion when the elbow is held at a weight-bearing angle (120–140°) (**A**,**B**). When the joint is extended, the anconeal process displaces more proximally into the supratrochlear foramen revealing the partial thickness cartilage lesion on the caudal aspect of the humeral condyle (**C**,**D**).

The dog was anaesthetised on the following day and bilateral elbow arthroscopy using a 2.4 mm, 30° oblique arthroscope (Arthrex, Munich, Germany) was performed using a recently described caudal arthroscopic portal [9]. On the left side, it confirmed the presence of a wide irregular midsagittal defect of the articular cartilage of the humeral condyle compatible with a fissure. The cartilagineous lesion previously reported in all Spaniels presenting with HIF was present on the caudo-proximal aspect of the humeral condyle, medial to the midline of the humeral condyle, and it presented as a focal (~1–2 cm in diameter) partial thickness cartilage damage. Intra-operative manipulation of the elbow joint to a weight-bearing angle (120–140°) confirmed that the tip of the anconeal process “engaged” exactly the cartilaginous lesion. The medial coronoid process and the rest of the articular surface of humerus and ulna appeared in normal condition. Arthroscopy of the right elbow revealed similar changes except for the intracondylar fissure that appeared to be deeper than on the left elbow (Figure 1).

**Figure 2:**
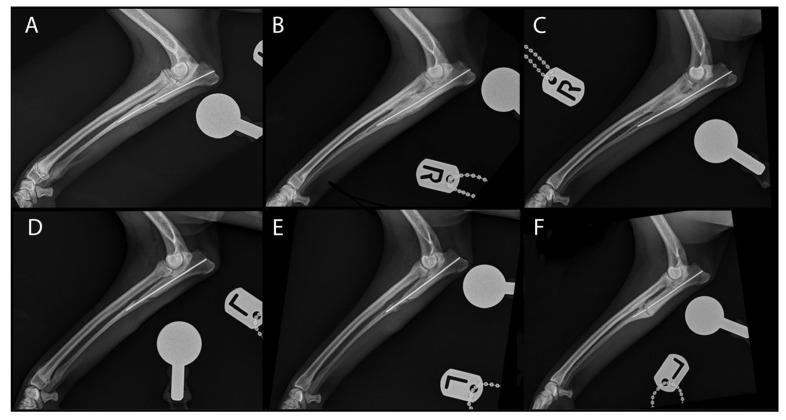
Medio-lateral view of the right and left antebrachium immediately after surgery (**A**,**D**), at the 5-week follow-up appointment (**B**,**E**) and at the 8-month follow-up appointment (**C**,**F**).

An oblique proximal ulnar osteotomy was therefore performed on the right thoracic limb as previously described [12]. The interosseous ligament was released by directing a Freer periosteal elevator proximally, on the cranial aspect of the ulna, and by application of a force in a medial direction until the ligament was transected. The periosteal elevator was then pulled distally until the ligament was completely released from proximal to distal. A 0.8 mm intramedullary K-wire was placed into the ulna in a retrograde fashion to prevent excessive caudal displacement of the proximal ulnar segment (Figure 2).

A 0.5 mL (equivalent to 0.75 mg of dibotermin alfa) of reconstituted bone morphogenic proteins (BMP) (InductOs, Medtronic BioPharma, Heerlen, The Netherlands) was uniformly distributed on a collagen haemostatic matrix (Lyostypt, B. Braun Medical, Sheffield, UK) which was subsequently applied at the osteotomy site with the aim to stimulate bone healing. A compressive bandage was applied for 3 days to limit post-operative swelling. 

Postoperative analgesia was provided by the administration of methadone (0.2 mg/kg intramuscular, every 4 h) (Comfortan, Dechra, Shrewsbury, UK) whilst in the hospital and meloxicam (0.1 mg/kg SID orally) (Loxicom, Norbrook, Corby, UK) for two weeks. Cage rest and lead-only walk for 10 weeks were instructed at discharge. 

Orthopaedic examination of the right elbow performed five weeks later revealed that discomfort on elbow extension had fully subsided whilst, severe discomfort was still present on extension of the left elbow. Follow-up radiographs of the right antebrachium demonstrated complete bone healing of the ulnar osteotomy with radio-ulnar synostosis present at level of the osteotomy site. An oblique proximal ulnar osteotomy was subsequently performed on the left side. As previously performed on the contra-lateral limb, the interosseous ligament was transected, and a 0.8 mm intramedullary pin was placed to avoid excessive caudal displacement of the proximal ulnar segment. Due to the degree of synostosis detected in the right antebrachium, BMP was not used on the left ulna. Use of a bandage, postoperative analgesia, and exercise restrictions were implemented as previously described.

## 3. Results

Follow-up examination performed five weeks postoperatively, revealed that discomfort on elbow extension had completely resolved bilaterally. Radiographs of the left antebrachium confirmed progressive bone healing at level of the osteotomy site without formation of obvious synostosis between radius and ulna as previously noticed on the contra-lateral limb. Radiographs of the right antebrachium confirmed presence of a complete mature callus osseous at level of the ulnar osteotomy site with complete radio-ulnar synostosis of the proximal quarter of the radius.

**Figure 3:**
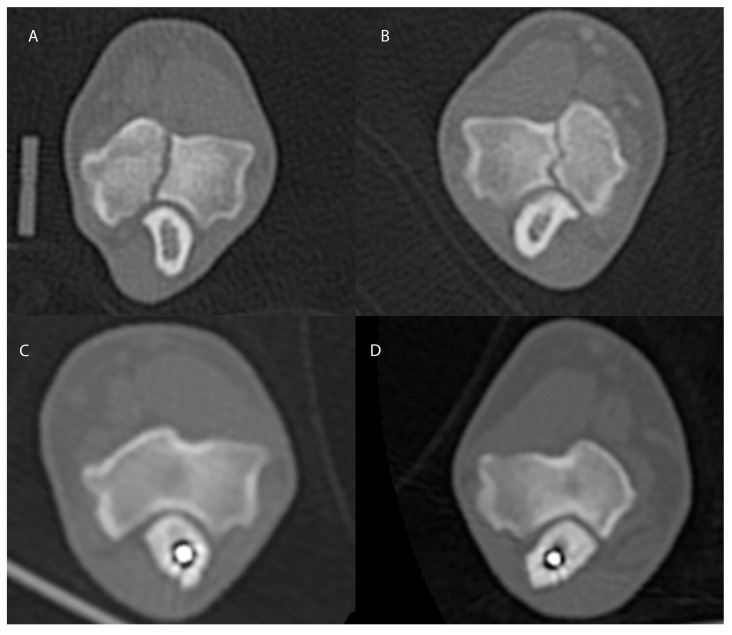
Coronal computed tomographic images of the left (**A**,**C**) and right (**B**,**D**) elbows. An obvious hypoattenuating line is present in the pre-operative images (**A**,**B**) representing a complete humeral intracondylar fissure extending from the caudal to the cranial articular surfaces. The fissures appear to have completely resolved in the CT images obtained 8 months after surgery (**C**,**D**).

Follow-up re-examination was performed eight months later; the dog was sound at walk on the thoracic limbs and orthopaedic examination revealed no discomfort on bilateral elbow manipulation. The patient was sedated, and CT-scan of both elbows was performed as previously described. The 3-D multiplanar reconstruction demonstrated complete healing of the HIF line and resolution of bone sclerosis surrounding the fissure line. (Figure 3) 

Radiographs of the right antebrachium were unchanged compared to the previous study and radiographs of the left antebrachium demonstrated that, despite 3 out of 4 cortices at the osteotomy site were bridged, signs of delayed union and pin breakage at the level of the osteotomy line were evident.

## 4. Discussion

This report describes the successful treatment of HIF in a Shetland sheepdog by performing a proximal ulnar osteotomy. To our knowledge, this is the first of its kind, both because of the unusual breed and because of the alternative treatment which has never been considered before for this disease. 

Humeral intracondylar fissures have so far been reported in Spaniel breed dogs, Tibetan mastiff, Yorkshire terrier, German shepherd, Lurcher, French bulldog, Pug, English pointer, Rottweiler, Labrador retriever, Giant schnauzer, German wirehaired pointer, Bavarian mount hound and German shorthaired pointer [2,3,4,13,14,15,16,17].

The initial hypothesis on the aetiology of HIF was that it was due to a failure to fuse of the two humeral condylar centres of ossification [3]. However, this theory has been challenged by three cases that more recently reported either formation of a new fissure or a partial fissure propagation to complete in an interval between two episodes of advanced imaging [6,18,19]. These reports corroborated the hypothesis that HIF in adult dogs is the result of a stress fracture and whilst we acknowledge that this may not be the case in younger dogs (as a different aetiopathogenesis of HIF may be present in a younger population of dogs), in our case pre-operative radiographs confirmed the physes of the distal humeri to be completely fused, making the incomplete ossification of the humeral condyle theory less likely. 

Fatigue fractures, sometimes equated to the term “stress fractures”, are the result of abnormal repetitive load on a normal bone. In humans, stress fractures occur mainly in the lower extremities and in the pelvis and they all share common pathognomonic appearance such as linear sclerosis, focal endosteal or periosteal reaction and fracture through one cortex with superimposed periosteal reaction [20]. The hypoattenuated line present through the humeral condyle is usually surrounded by intense sclerosis and new bone formation/periosteal reaction of the lateral epicondylar crest that can be present in up to 80% of cases [21]. The same study revealed that in those cases where an incomplete HIF was diagnosed by CT scan, the fissure was subjectively considered to originate from the articular surface at the most caudo-dorsal aspect of the humeral condyle [21]. Although sequential CT studies are needed to confirm this finding, one could speculate that HIF may develop as a consequence of the humero-anconeal incongruity that is affecting the caudal portion of the joint.

An increase in frequency, duration, or intensity of an abnormal repetitive load on normal bone will lead to plastic deformity and then a stress fracture [20]. Similarly, cyclical loading applied by the anconeal process to the caudal aspect of the humeral condyle during the weight bearing phase could result in cumulative bone strain leading to bone damage and fracture if net bone damage exceeds bone repair (when bone resorption induced by osteoclasts is greater than bone replacement induced by osteoblasts). This hypothesis is supported by a recent publication in dogs with HIF that described the tip of the anconeal process perfectly matching the focal cartilagineous lesion only when the elbow was positioned at a weight-bearing angle. Treatment of stress fractures in humans is generally conservative because of the lesser risk of complications and protected or limited weight bearing is recommended for periods up to 6 months to allow for the normal bone turnover to be re-established [20]. We propose that performing a proximal ulnar osteotomy in a dog suffering from HIF due to humero-anconeal incongruity may prevent the anconeal process from applying this abnormal load to the humeral condyle, allowing re-establishment of the correct balance between osteoblastic and osteoclastic activity of the bone, and therefore encouraging natural healing of the fissure. This theory has recently been supported by the preliminary results of a study involving the use of proximal ulnar osteotomy as a treatment of HIF in Spaniel breed dogs which proved that complete healing of the fissure was achieved in 60% of elbows, partial healing in 26.6% and lack of healing in 13.3% [22].

The standard procedure for surgical management of HIF involves placement of a transcondylar screw in a lag or positional fashion. The aim is to minimise the movement between the medial and lateral condyles in order to prevent fracture development and to improve lameness. The reported overall complication rate following transcondylar screw placement is between 8 and 59.5% with the most commonly reported complications being seroma formation and surgical site infection [4,7,13,23]. Implant failure in the form of screw breakage and aseptic loosening has been reported in 2.5–10% of the cases treated with transcondylar screw [7,8,21,23]. Persistent lameness following screw placement has also been reported in one study, with 23% and 30% of dogs reported to require respectively sporadic or daily analgesia at the time of the last follow-up [23]. By avoiding placing a transcondylar screw, we avoided all the aforementioned type of complications that can sometimes lead to catastrophic consequences. However, a proximal ulnar osteotomy is not a procedure free of complications and despite the fact that these are usually more benign and easier to address from a surgical point of view, the reported overall complication rate for this procedure can vary from 13–54%. These include excessive proximal segment caudal migration, delayed osteotomy union, infection, seroma formation, haemorrhage and radial head subluxation [12,24,25,26]. In our case, delayed union of the osteotomy was observed on the left ulna at the 8-month follow-up appointment; this is likely due to breakage of the intramedullary pin after the 5-week radiographic follow-up appointment was performed. This led to excessive motion at the osteotomy site and formation of a larger than usual callus osseous without jeopardising the dog’s recovery. Proximal ulnar osteotomy is a surgical technique used as a treatment for elbow joint incongruency [10,11,26]. Following a PUO, the proximal ulnar segment moves in a complex multi-directional way and settles in a position that is mainly influenced by the soft tissue attachments, the articular surface and the loading forces. Proximal translation, varus deformity, caudal tipping and axial rotation will allow the anconeal process to move in a more cranio-proximal direction towards the supratrochlear foramen [27]. This change in position of the anconeal process should therefore interrupt the application of an abnormal force against the caudal aspect of the humeral condyle and may allow physiologic healing of the stress fracture. Healing of the HIF has been inconsistently reported in the veterinary literature and, currently, no techniques seem to promote consistent healing. Use of allograft or autograft in combination with a strong implant fixation have been recommended to manage these challenging non-healing stress fractures [28]. However, there is no objective and quantified method to report healing of the fissure as the use of advanced imaging (to assess post-operative healing of the fissure) is limited by the presence of the transcondylar screw and by the artefacts it generates. In our case, absence of implants allowed us to document complete healing of the HIF in both elbows and resolution of the associated sclerosis and whilst we acknowledge that additional studies are needed to confirm that proximal ulnar osteotomy is the cause of the healing of the HIF, we can speculate that this is the case as we are not aware of any scientific report documenting spontaneous healing of the HIF in an almost skeletally mature dog.

We elected to use BMP during the first surgery to promote bone union of the two ulnar segments as were concerned about excessively slow healing of the ulna and risk of delayed/non-union. This led to a significant degree of synostosis between the proximal radius and ulna. In humans, congenital and acquired proximal radioulnar synostosis can cause functional deficits due to a reduced range of pronation-supination [29,30]. In young dogs, restriction of movement between radius and ulna can result in a noticeable alteration of the growth characteristics and of the morphology of the bones. However, the radiographic changes associated with the acquired synostosis depend on the age of the acquired event and synostosis between radius and ulna in the mature animal and do not result in deformity [31,32]. Despite the fact that our dog had not reached skeletal maturity and the radial and ulnar physes were still active at the time of performing the initial surgery, it did not seem to affect the normal growth of radius and ulna and it should therefore not lead to future major functional deficits. 

We acknowledge several limitations in this report, the most important of which is the fact that it includes only a single case, and more cases and an appropriate control group are needed to prove if PUO can reliably allow physiologic healing of the fissure. The second limitation is the lack of a control group as it is unknown if the HIF could have healed with no surgical treatment or by simple placement of a transcondylar screw. Lastly, second-look arthroscopy to confirm healing of the cartilaginous lesion present on the caudal aspect of the humeral condyle would have been necessary to confirm the complete resolution of humero-anconeal incongruency, but it was considered an unnecessarily invasive procedure and it was therefore not performed.

## 5. Conclusions

To the authors’ knowledge, presence of a humeral intracondylar fissure in a Shetland sheepdog has not previously been described. Healing of the HIFs of both elbows was achieved after performing a bilateral staged proximal ulnar osteotomy.

## Data Availability

Not applicable.
